# Telediagnosis performance of specialists in oral medicine and general dental practitioner using images of oral mucosa lesions in Chile

**DOI:** 10.4317/medoral.26825

**Published:** 2024-11-25

**Authors:** Constanza Morales-Gómez, Gabriel Ojeda-Uribe, Daniela Adorno-Farías, Andrea Maturana-Ramirez, Iris Espinoza-Santander

**Affiliations:** 1 Departament of Pathology and Oral Medicine, Faculty of Dentistry, University of Chile, Santiago, Chile

## Abstract

**Background:**

The objective of this research was to evaluate the performance of specialists in oral medicine doing diagnosis of oral mucosa lesions through digital images comparing general dental practitioner in Chile.

**Material and Methods:**

20 oral medicine specialists from the national registry of specialists in Chile and 20 general dental practitioners were invited to participate. Each participant reviewed 33 cases with digital images of oral mucosa lesions and was asked to submit diagnostic hypotheses. The proportions of correct diagnoses and diagnostic accuracy were determined. Analyses were performed using STATA 16.0.

**Results:**

Specialists presented a higher total proportion of correct diagnoses than general dental practioners (86.5% vs 49.2%). Specialists also showed higher sensitivity (88.5% vs 59.3%) and greater specificity (85.8% vs 48.6 %) than general dental practioners in the diagnosis of oral cancer and oral potentially malignant disorders compared to benign oral lesions.

**Conclusions:**

In Chile, oral medicine specialists are a reliable alternative to provide diagnostic guidance through e-consult, but its margin of error must be considered.

** Key words:**E-consult, oral mucosal lesions, teledentistry, telediagnosis, oral medicine.

## Introduction

Electronic consulting or interconsultation (e-consult) is a telemedicine or teledentistry modality that encompasses the exchange of information, clinical images, opinions and tele-diagnostic guidelines and therapeutic recommendations on a clinical case. It occurs between members of the health team, without the presence or direct participation of the patient during this exchange. In the field of oral medicine, this modality has been proposed as a tool to improve the access to a specialist, contributing to the early diagnosis of oral and maxillofacial diseases for an adequate referral and timely resolution (1,2). High acceptance of this modality by general dental practitioner and specislits has been reported (3,4). Recently, several experience of teledentistry in oral medicine with alternative approach for remote screening, diagnosis, consultation and treatment has been published (3,5-7).

With the increase of teledentistry in oral medicine advantages and disadvantages must be considered. While teledentistry offers many benefits, including improved access to care and convenience, it is not without its limitations and the main are limited physical examination, technological barriers, lack of hands-on procedures (example diascopy), data security, privacy concerns, loss of personal connection and legal regulatory changes. Critical to recommending is having adequate research on the diagnostic capacity of the specialists who use it but reports on the percentage of correct diagnoses (6-8) and sensitivity and specificity (9,10) are relatively scarce.

As the utilization of teledentistry in oral medicine continues to rise, it becomes imperative to delve into these aspects through research. By conducting comprehensive studies, the dental and oral health community can unlock the full potential of telediganosis in oral medicine, thereby elevating patient care and enhancing oral health outcomes. For this reason, the objective of this research was to evaluate the performance of specialists in oral medicine in Chile doing diagnosis of oral mucosa lesions through digital images comparing general dental practitioner.

## Material and Methods

This was an analytic cross-sectional study. The proportion of correct diagnosis and diagnostic accuracy of oral medicine specialists and general dental practitioners examinating oral mucosa lesions through digital images were evaluated. To calculate the sample size, an estimated proportion of correct diagnoses was used: 90% for specialists and 50% for general dental practitioners, with a significance level of 0.05 and statistical power of 0.8. The oral mucosa lesions images were evaluated by 20 specialists randomly selected from the Chilean National Register of individual health providers (September, 2022) and 20 general dental practitioners who worked in Primary Health Care Centers. Only one specialist declining to participate and she was replaced. The inclusion criteria were one year of work experience at least.

33 consecutives cases of patients with oral mucosa lesions with complete clinical and sociodemografic information that were registered in the Oral Medicine Clinic at the Faculty of Dentistry of the University of Chile were selected (Supplement 1). All these cases had one or two clinical images obtained with extraoral digital photographic cameras. The cases selected had relevant clinical information such as age, sex, reason for consultation, clinical diagnosis, and the pathology report, when it was available for the diagnosis. Clinical cases in which the face of the patient appeared in the photographs, and could identify them were excluded from the study. The oral mucosa lesions had diverse etiology: three cases of oral cancer (squamous cell carcinoma), four oral potentially malignant oral disorders (lichen planus, leukoplakia, proliferative verrucous leukoplakia), and 26 reactive and other benign lesions (irritative fibroma, mucocele, pyogenic granuloma, peripheral ossifying fibroma, traumatic ulcer, nevus, macule melanotic, amalgam tattoo, hemangioma, foliate papillary hyperplasia, pleomorphic adenoma, fibrolipoma, squamous papilloma, condyloma acuminatum, pemphigus vulgaris, recurrent oral ulcers, angular cheilitis, denture stomatitis, erythematous candidiasis).

- Procedure for the evaluation of digital images of lesions of the oral mucosa

The 33 selected cases were organized with a brief medical history and their clinical images, and were uploaded to a Google Form. Prior to this, the form was validated in terms of comprehension and language by three specialists in oral medicine from the research team, as well as three general dental practitioners.

An appointment was scheduled with each participant to evaluate the cases. The participants reviewed the cases either in a face-to-face meeting or in a synchronous videoconference using the Zoom® platform. Each consultant was asked to provide a maximum of two diagnostic hypotheses for each case, following the methodology used by Torres-Pereira *et al*. (7).

- Statistical analysis

An analysis of the results was conducted using descriptive statistics with proportions. The following analyses were performed:

1) Proportion of correct diagnoses between the diagnostic hypotheses provided by the participants and the gold standard (histopathology report for 22 cases or clinical diagnosis by the specialists from the research team for 11 cases).

2) Diagnostic accuracy: This refers to the level of accuracy with which participants correctly diagnosed oral cancer/ oral potentially malignant disorders in comparison to the other diagnoses (benign lesions). The parameters of sensitivity and specificity were used to describe the diagnostic accuracy.

The statistical significance analysis regarding the proportion of correct diagnoses between both groups was conducted using the simple proportion test. All analyses were performed using the STATA 16.0 Statistical Program, and *p-value*s ≤ 0.05 were considered significant.

- Ethical considerations

This study has been approved by the Ethics Scientific Committee of the North Metropolitan Health Service in February 2021 (Number 007/2021) to protect interest of human subjects in research. All study participants, including general dental practioners and oral medicine specialists, participated voluntarily and provided signed informed consent, understanding that they had the right to withdraw from the research at any time. The confidentiality and privacy have been strictly maintained and respected in accordance with the principles outlined in the Declaration of Helsinki for research involving human subjects.

## Results

20 oral medicine specialists from the national registry of specialists in Chile and 20 general dental practitioners completed the study. Only one specialist refuse to participate in the study, so an additional specialist was invited to participate. 55% of the oral medicine specialists were women and 45% among the general dental practitioners.

Specialists in oral medicine had a higher overall proportion of correct diagnostic outcomes compared to general dental practitioners, and the difference was statistically significant (*p*=0.01). When grouping the oral mucosal lesions into "oral cancer/oral potentially malignant disorder" and "reactive/other benign lesions," we observed that specialists in oral medicine achieved a higher proportion of correct diagnoses in both groups of lesions (Table 1).

**Figure 1:**
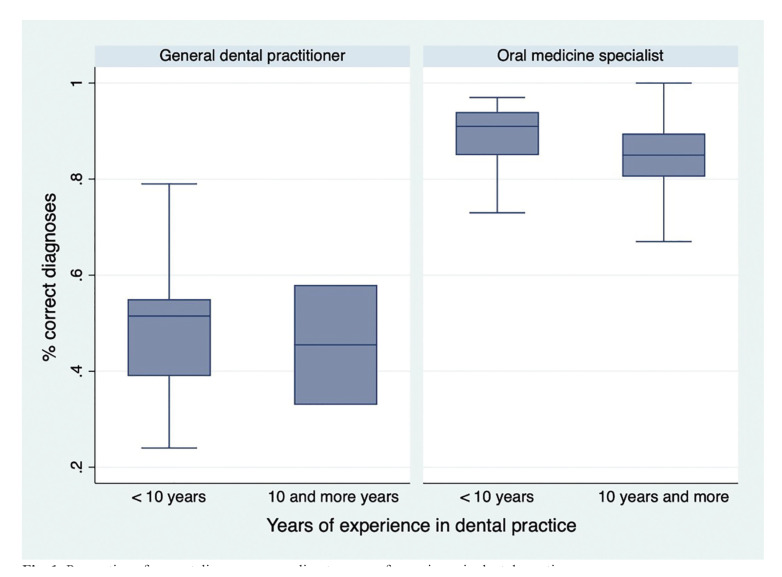
Proportion of correct diagnoses according to years of experience in dental practice.

The average of experience in dental practice (years since obtaining the professional title of dentist) were 16,3 (min-max; 5-46) in specialists and 5,9 (min- max; 1-24) in the general dental practitioners. Disaggregated according to years of experience in dental practice (<10 years, and 10 years and more), it is observed that specialists have a greater number of correct diagnoses independent of the number of years (Fig. 1).

Considering each one of the lesions included in this study, we observed that in seven cases all of the oral medicine specialists achieved a correct diagnosis (100%). These cases were squamous cell carcinoma (case 27), oral leukoplakia (case 13), actinic cheilitis (case 22), angular cheilitis (case 24), pleomorphic adenoma (case 12), melanotic macule (case 26), and mucocele (case 17).

In the group of general dental practitioners, no cases achieved correct diagnosis by all participants. The 6 lesions with the highest proportion of correct diagnosis were: 95% angular cheilitis (case 24), 90% irritative fibroma (case 5), 85% leukoplakia (case 13), 80% proliferative verrucous leukoplakia (case 19), 80% mucocele (case 17), 80% mucocele (case 25), and 80% melanotic macule (case 26). The complete Table of the proportion of correct diagnosis in all the cases is shown in Table 2.

For oral medicine specialists, the diagnostic accuracy for oral cancer/oral potentially malignant disorders with respect to reactive/benign lesions showed a sensitivity of 88.6% and specificity of 85.8%. In the case of general dental practioners, the sensitivity was 59.3% and the specificity was 46.5%. The values and confidence intervals for these results can be seen in Table 3.

## Discussion

Studies on the performance of specialists in oral medicine and general dental practitioners regarding remote diagnosis of oral mucosal lesions using digital images are scarce (6,7,8,11) and have not been previously conducted in Chile. However, it is important to develop such studies due to the increasing implementation of e-consultation in oral medicine in different countries (12). In Chile, recently, since June 2020, an e-consultation strategy in oral medicine has been implemented, available to primary health care dentists (12). In our research, specialist in oral medicine show a high performance, with cases in which all specialists reached the correct diagnosis, and better diagnostic acurracy than general dental practitioners.

Strengths of this study are the inclusion of oral mucosal lesion images of diverse etiology, representing the spectrum of common consultations in the dental field of this specialty. Additionally, a nationally representative sample of specialists was selected, obtained randomly from the national registry of providers and from different geographical regions of Chile. In terms of the total number, these specialists accounted for a high percentage of the national total, 20 out of 62 registered as of September 2022 (32.2%). Moreover, there was a high level of acceptance of participation (95%) with only one specialist declining to participate. Another strength is that, in addition to specialists, this study includes general dental practioners as a comparison parameter. This aspect was not considered in other studies, which solely focused on the abilities of specialists. Regarding to the number of participants, in this study 20 specialists were evaluated, whereas in other studies, it was only two (6,7) or three specialists (8).

As a limitation, we must mention that the sample of general dental practioners was not randomized due to the unavailability of contact information to invite them to participate. Nevertheless, dentists from different primary health care centers in the Metropolitan area (where 45% of the national population resides) were included. Additionally, other important limitation is the lower years of experience mean in dental practice of the general dental practitioners compared to oral medicine specialists. This is associated with the fact that oral medicine specialists have worked several years previously as general dental practitioner prior to their specialization program studies.

General dental practitioners achieved a 49.2% of proportion of correct diagnoses, which was lower than that of the specialists. Different studies in general dental practitioners have reported that this group acknowledges the need for more training and experience in recognizing oral lesions, including oral cancer and other oral mucosal lesions (13,14). Self-perceived ability for diagnosing these lesions is low among general dental practitioners, and they do not feel adequately prepared to perform such diagnoses within their professional practice. Consequently, they rely on the referral of patients to other specialists (13). This ability has been associated to the number of years of professional practice (15).

On the other hand, specialists achieved a 86.5% proportion of correct diagnoses in oral mucosal lesions, much higher than that of general dental practioners. Although it had not been quantified previously, this result was expected. This group is highly trained in diagnosing and treating these lesions and can recognize different presentations of the same disease (16). In Chile, the specialization program has been offered since 1996, currently in three national universities.

Regarding the proportion of correct diagnoses by specialists to compare with our results, there are limited reports. A previous study conducted in Brazil, which included only two participating specialists, observed proportions of correct diagnoses of 63.3% and 70% (7), which were lower than those obtained in our study. Petruzzi and Benedittis (11) found 82% of proportion of correct diagnoses regarding clinicopathologic evaluation analyzing images taken by patients, dentists, physicians, or dental hygienists with their personal smartphones and sent by WhatsApp to oral medicine specialists. However, these values cannot be directly compared because they differ in the types of lesions included. Additionally, the clinical images used in that study were not included in these articles, making it impossible for us to assess the image quality and the level of difficulty of the selected clinical presentations.

Determining whether our results regarding the difference in diagnostic accuracy in oral mucosa lesions diagnosis between specialists and general dental practioners are above or below the standard is not possible because there are no studies that have previously compared these groups. This could be evaluated in future research in other contexts.

In the medical field of dermatology, performance has been evaluated in several studies ranged the proportion of correct diagnosis from 48% to 91% (17-22). Two studies that assessed malignant and benign circumscribed cutaneous lesions, such as basal cell carcinoma, seborrheic keratosis, dermal nevus, among others, found diagnostic agreement rates of 64% (19) and 65% (20). In the diagnosis of pigmented and non-pigmented skin lesions, telemedicine is a tool that meets accepTable standards, achieving an 80% accuracy rate (23).

In terms of diagnostic accuracy, our results are consistent with previous teledermatology studies where sensitivity values have been reported to range from 88% to 100%, and specificity values between 39% and 98% (18,24,25).

Our findings reinforce the idea that teledentistry, through e-consult, is a valuable tool for providing diagnostic guidance when the professional possesses the necessary expertise in the relevant field (26). In this case, specialists in oral medicine in Chile demonstrated greater competence in providing guidance on oral mucosal lesions than general dental practitioners. This result allows us to recommend incorporating this activity into the professional practice of these specialists as an alternative to improving access to specialized care, especially in areas where the number of specialists is limited. By doing so, the opportunity for rapid evaluation and triage can be increased, benefiting individuals in rural areas. It is important to consider that incorporating this new mode of care does not entail eliminating in-person clinical care, as certain elements of the clinical examination, such as medical history, palpation, and dentist-patient communication, remain irreplaceable pillars of oral medicine care and are essential for treatment continuity. However, the use of this tool undoubtedly improves the relevance and referral to the speciality in oral medicine.

## Conclusions

Oral medicine specialists in Chile demonstrated strong performance in identifying oral mucosal lesions through digital images and showed greater diagnostic abilities than general dental practitioners, supporting the incorporation of telediagnosis into their professional activities.

## Figures and Tables

**Table 1:** Proportion of correct diagnoses in oral mucosa lesions by oral medicine specialists and general dental practitioners.

	Total percentage of correct diagnoses % (min-max)	Oral cancer and oral potentially malignant disorders correct diagnoses %(min-max)	Reactive lesions and other benign lesions correct diagnoses %(min-max)
Oral medicine specialists	86,5 (67-100)*	88,6 (40-100)*	85,8 (55-100)*
General dental practitioners	49,2 (24-79)	58,6 (25-85)	46,5 (5-95)

*=*p value* ≤ 0,05.

**Table 2:** Proportion of correct diagnoses by oral medicine specialists and general dental practitioners for each clinical case.

	Diagnosis	N° Case	Oral medicine specialist %	General dental practitioner %	*p value*
Oral cancer	Squamous cell carcinoma	14	40	25	0,311
		16	95	45	< 0,05
		27	100	60	< 0,05
Oral potentially malignant oral disorders	Lichen planus	7	95	65	<0,05
	Leukoplakia	13	100	85	0,072
	Proliferative verrucous leukoplakia	19	90	75	0,212
	Actinic cheilitis	22	100	55	< 0,05
Reactive lesions and other benign lesions	Irritative fibroma	1	85	30	< 0,05
		2	95	80	0,152
		5	90	90	1,000
	Peripheral ossifying fibroma	3	55	15	< 0,05
	Mucocele	6	75	65	0,490
		17	100	80	< 0,05
		25	75	80	0,705
	Pyogenic granuloma	10	80	30	< 0,05
		20	65	25	<0,05
		33	95	5	<0,05
	Traumatic ulcer	29	85	25	<0,05
	Nevus	9	95	50	< 0,05
	Melanotic macule	26	100	80	< 0,05
	Amalgam tattoo	11	90	40	< 0,05
	Hemangioma	23	95	45	< 0,05
	Foliate papillary hyperplasia	32	80	15	< 0,05
	Pleomorfic adenoma	12	100	20	< 0,05
	Fibrolipoma	30	65	50	0,337
	Squamous papilloma	21	75	40	< 0,05
	Condyloma acuminatum	15	80	55	0,091
	Pemphigus vulgaris	8	85	20	< 0,05
		31	95	5	< 0,05
	Recurrent oral ulcers	4	95	65	< 0,05
	Angular cheilitis	24	100	95	0,311
	Denture stomatitis	28	80	70	0,465
	Erythematous candidiasis	18	95	35	< 0,05

**Table 3:** Values of sensitivity and specificity in the recognition of oral cancer/ oral potentially malignant disorders.

	Sensitivity %(min-máx)	95% confidence interval	Specificity %(min-máx)	95% confidence interval
Oral medicine specialists	88,6 (57,1-100)	82,9 - 94,1	85,8 (61,5-100)	81,4 - 90,2
General dental practitioners	59,3 (14,3-100)	48,6 - 69,9	46,5 (23,1-76,9)	39,9 - 53,2

## Data Availability

The authors confirm that the data supporting the findings of this study are available within the article and its supplementary materials.
